# Exploiting Viscoelastic Experimental Observations and Numerical Simulations to Infer Biomimetic Artificial Tendon Fiber Designs

**DOI:** 10.3389/fbioe.2019.00085

**Published:** 2019-05-07

**Authors:** Nikolaos Karathanasopoulos, Jean-Francois Ganghoffer

**Affiliations:** ^1^Chair of Computational Modeling of Materials in Manufacturing, ETH Zürich, Zurich, Switzerland; ^2^LEM3, CNRS, University of Lorraine, Metz, France

**Keywords:** tendon, tissue engineering, biomaterials, viscoelasticity, fibers, relaxation, matrix

## Abstract

Designing biomimetic artificial tendons requires a thorough, data-based understanding of the tendon's inner material properties. The current work exploits viscoelastic experimental observations at the tendon fascicle scale, making use of mechanical and data analysis methods. More specifically, based on reported elastic, volumetric and relaxation fascicle scale properties, we infer most probable, mechanically compatible material attributes at the fiber scale. In particular, the work provides pairs of elastic and viscous fiber-scale moduli, which can reproduce the upper scale tendon mechanics. The computed range of values for the fiber-scale tendon viscosity attest to the substantial stress relaxation capabilities of tendons. More importantly, the reported mechanical parameters constitute a basis for the design of tendon-specific restoration materials, such as fiber-based, engineering scaffolds.

## Introduction

**Figure 1:**
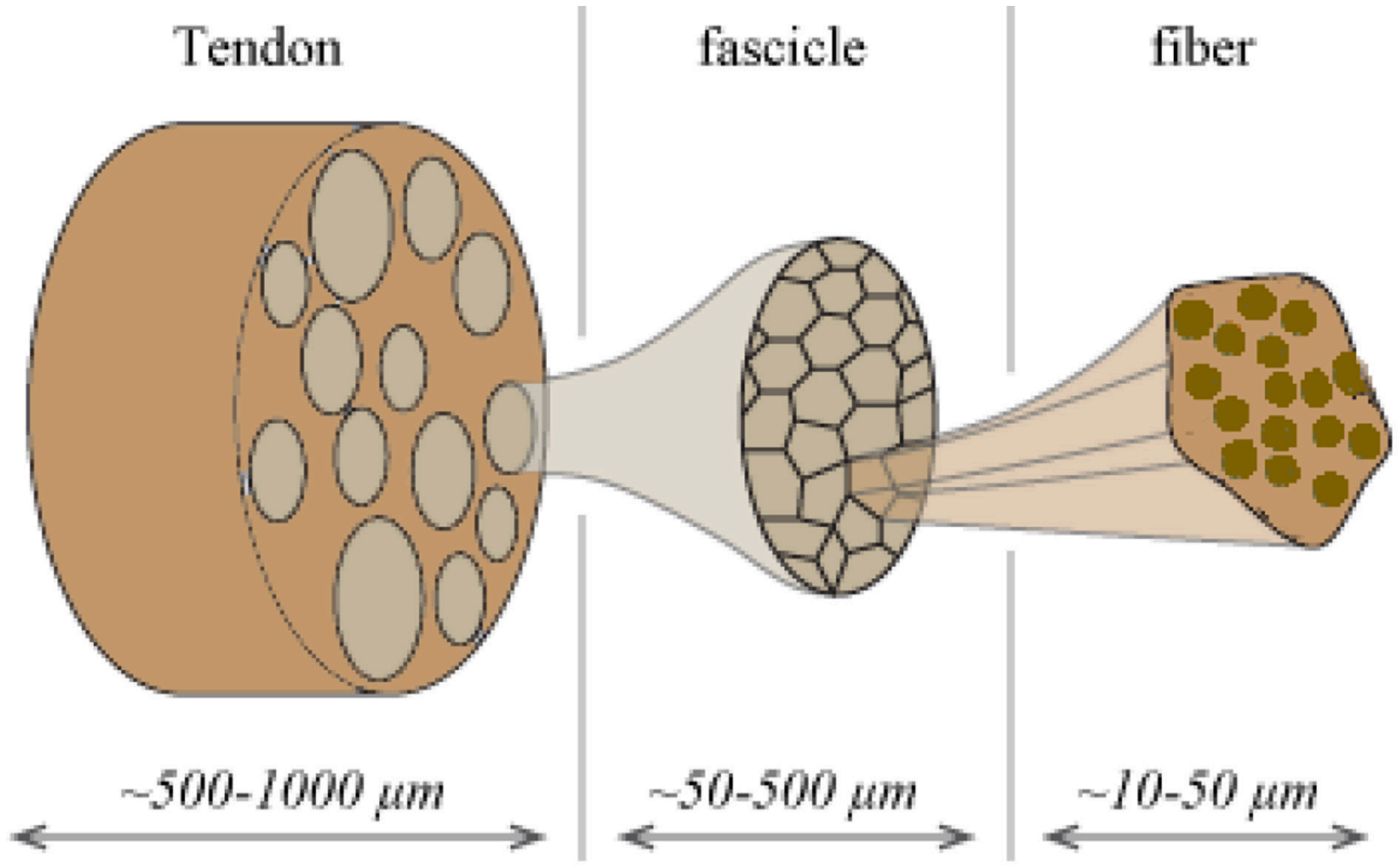
Schematic representation of the tendon's hierarchical inner structure: fibers are enclosed into fascicles which form the tendon macrostructure.

Tendons are natural fibrous tissues that transfer mechanical loads from the muscles to the bones. They are structured in a highly hierarchical manner (Maceri et al., 2012), consisting of a series of inner fibrillar scales that are immersed in a matrix substance (Shen, 2010; Zhang et al., 2014). As for their structural arrangement, both human and animal tendon specimens have been commonly described as a multiscale composite materials. The tendon unit consists of fascicles (at the scale of hundreds of micrometers), which are in turn composed of matrix-immersed fibers (micrometer). Figure 1 provides a schematic representation of the tendon's inner multiscale architecture (Figure 1) (Goh et al., 2008; Thorpe et al., 2014).

The tendons' inner fibrillar components are not parallel to the length evolution of the tendon. On the contrary, they are structured in helical patterns (Orgel et al., 2006), forming an undulated inner structure; an observation reported in different microscopy based studies (Yahia and Drouin, 1989; de Campos Vidal, 2003). Their helical arrangement, with a typical angular range in between 70 and 76° with respect to the plane normal to the tendon axis evolution (Järvinen et al., 2004; Starborg et al., 2013), leads to a characteristic coupled axial and torsional behavior at the fascicle scale (Thorpe et al., 2013). The effective mechanical behavior at the tendon macroscale, arises as a rather complex function of the properties of its inner constituents (Reese et al., 2013). The latter have been commonly measured by elastic, uniaxial strain experiments, carried out independently at the different inner tendon scales (Figure 1). At the lower scale of fibers, elastic moduli values of *E*_*f*_ = 0.57 ± 0.08 *GPa* and *E*_*f*_ = 2.69 ± 0.42 *GPa* have been reported for wet and dry rat tail tendon fiber specimens, respectively (Kato et al., 1989). Independent experimental studies provided moduli values of *E*_*f*_ = 1.17 ± 0.28 *GPa* for hydrated fiber tendon specimens (Gentleman et al., 2003), within the range reported by Kato et al. (1989). At the uppermost inner hierarchical scale of fascicles, experimental data suggest a substantially lower overall uncertainty. In particular, elastic moduli values of *E*_*fasc*_ = 0.64 ± 0.03 *GPa*, *E*_*fasc*_ = 0.48 ± 0.07 *GPa*, and *E*_*fasc*_ = 0.55 ± 0.14 *GPa* have been reported (Derwin and Soslowsky, 1999; Lavagnino et al., 2005; Haraldsson et al., 2009; Svensson et al., 2010), exhibiting a maximum difference of 0.2 *GPa* with respect to their mean value.

The overall decrease observed in the elastic moduli of the upper scales is to be primarily attributed to the presence of the non-collagenous matrix substance (Figure 1). The latter has a quasi-negligible stiffness contribution with respect to the one provided by the fibrillar components. While no direct experimental measurement is available, analytical computations have estimated a matrix modulus *E*_*m*_ below 1 MPa (Ault and Hoffman, 1992) that is more than two orders of magnitude lower than the one reported for any fibrillary component measurement (Thorpe et al., 2017); a value that has been typically employed in numerical studies (Reese et al., 2010). The content of fibers *f*_*r*_ within fascicles (Figure 1) varies with the tendon's age and health state (Lavagnino et al., 2005; Svensson et al., 2010). Relevant studies provide fibrillar contents *f*_*r*_ as low as 35% when a certain level of fiber swelling is accounted for (Svensson et al., 2012) and up to more than 60% (Hansen et al., 2010; Svensson et al., 2010).

The combination of high moduli differences amongst the embedding matrix and the fibrillar components (*E*_*f*_ >> *E*_*m*_), along with the helical arrangement of the tendon subunits have been shown to constitute the basic mechanisms responsible for the tendon's distinctive volumetric behavior (Reese et al., 2010; Swedberg et al., 2014; Karathanasopoulos et al., 2017). More specifically, experimental observations have reported Poisson's ratio values ν that well-exceed the ones observed in common engineering materials. In particular, mean Poisson's ratio values close to unity (Cheng and Screen, 2007) and up to 3 have been reported (Lynch, 2003), accompanied by substantial uncertainties.

**Figure 2:**
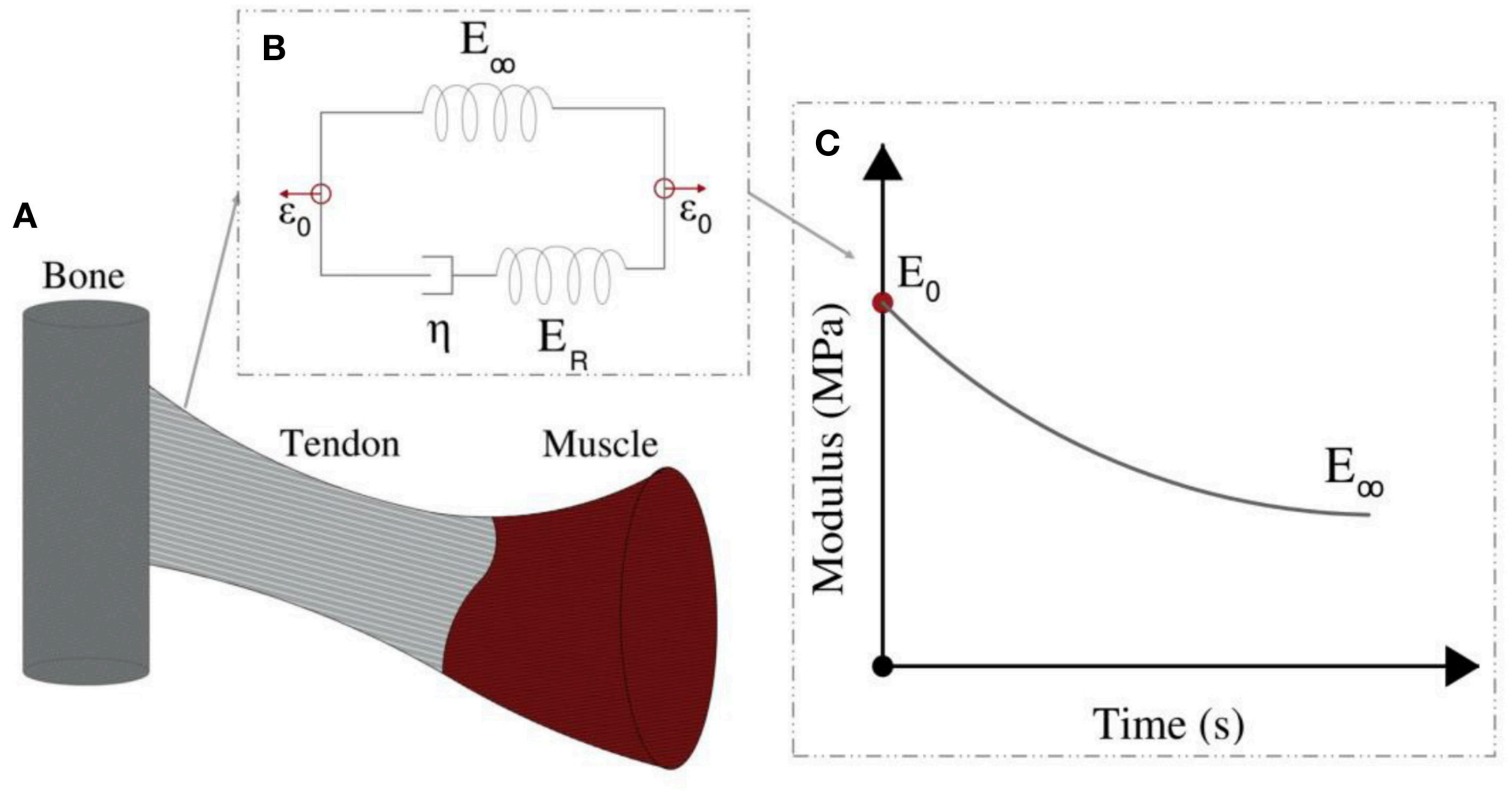
Tendons transfer forces from muscles to bones **(A)**. Their modulus depends on the loading time **(C)**. Their relaxation behavior can be represented by the Standard linear solid model **(B)**.

**Figure 3:**
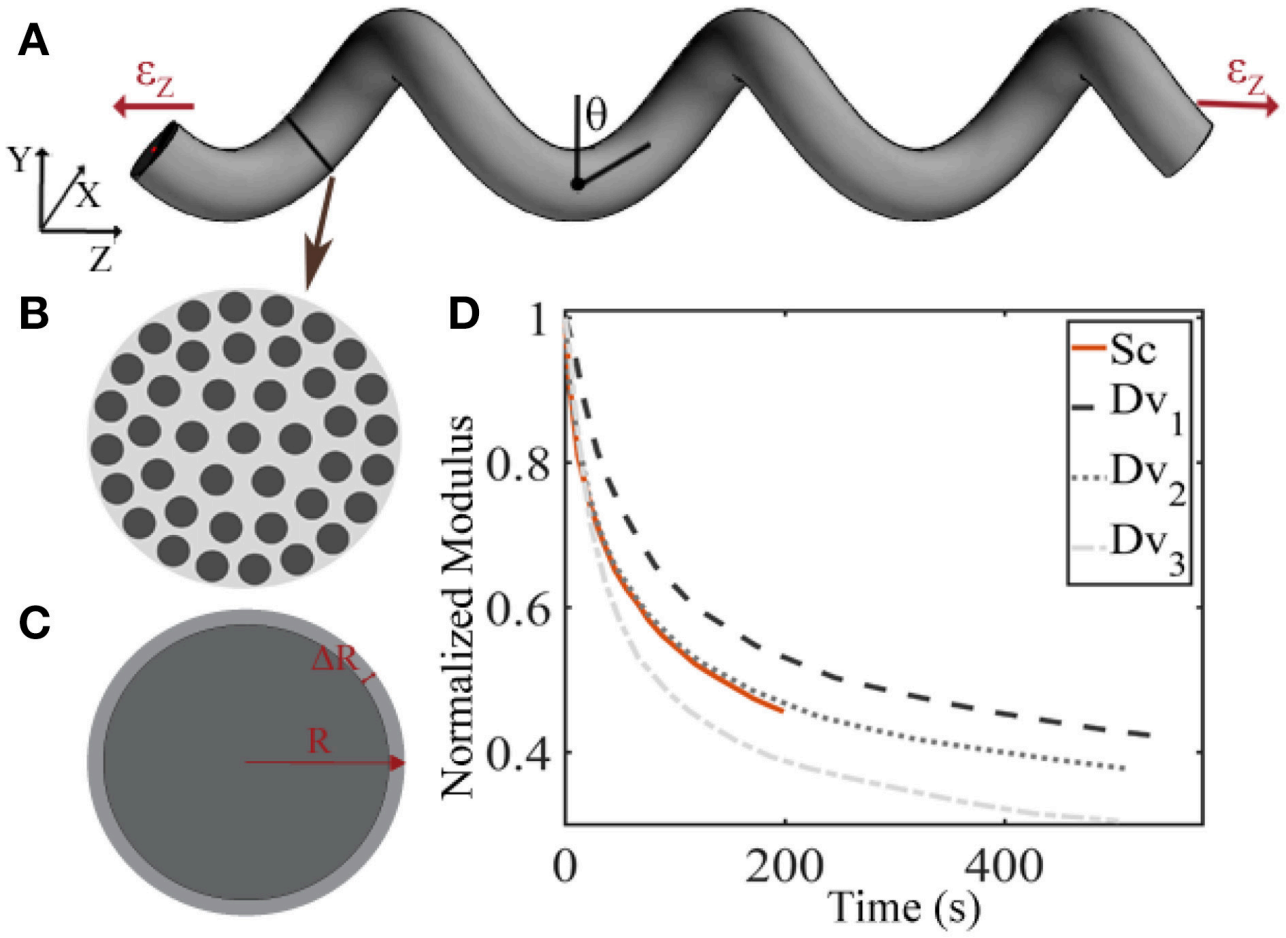
Schematic representation of the tendon fascicle's helical geometry **(A)** that is composed of matrix-embedded fibers **(B)** (Goh et al., 2008), with a Poisson's ratio behavior **(C)** that is dependent on the fascicle's structural composition **(B)** and geometry **(A)**. In subplot **(D)**, the tendon fascicle's experimental relaxation curves as provided by Screen (2008) (Sc) and Davis and De Vita (2012) (Dv) are summarized.

The linear elastic response cannot however explicate the considerable stress and shock absorption capacities of tendons (Salathe and Arangio, 1990), which are directly associated to a viscous, time-dependent behavior. The tendon's viscoelastic nature allows for the attenuation of stress stimuli and is commonly characterized by a viscosity parameter η, which is directly related to a relaxation time τ (Lakes, 2009), as schematically depicted in Figure 2. Tendon inner scales have been shown to yield a viscoelastic, time-dependent response, which however has not been typically characterized with respect to its effective viscosity value η. In particular, at the tendon fascicle scale, relaxation curves with a time-dependent modulus evolution over a range of 200 s and up to more than 500 s have been provided in independent experimental studies (Screen, 2008; Davis and De Vita, 2012) (Figure 3D). Relaxation experiments suggest a loss of 40–70% of the initial elastic modulus at the tendon fascicle scale over this time interval (Screen, 2008).

The material properties of the tendon building blocks considerably affect its functionality and its load transfer efficiency (Rawson et al., 2015); parameters of primal importance for connective tissues (Galbusera et al., 2014). A thorough quantification of the material properties of the tendons' inner constituents is essential, not only for the understanding of its nature (Balint et al., 2014), but most importantly for any repair and restoration process to take place (Robinson et al., 2004; Laurent et al., 2014; Lee et al., 2017). Up to now, restoration processes have primarily used biological and synthetic scaffolds (Kuo et al., 2010; Kwon et al., 2014; Sandri et al., 2016). Biological treatments employed biodegradable silk-collagen scaffolds that were meant to provide advanced regeneration capabilities (Abdullah, 2015). Synthetic replacements were based on textile scaffolds selected out of a list of existing substitutes (Lomas et al., 2015). However, scaffolds of the kind have been reported to show limited mechanical biocompatibility (Ratcliffe et al., 2015; Tang et al., 2018). Statistical data on the success of surgical restorations reveal rather low success rates. More specifically, for tendons injuries with large and extensive damage, the mean post-surgical, operation success rate does not exceed a mere 50 and 40%, accordingly (Meimandi-Parizi et al., 2013).

A part of the low efficiency of current restoration practices needs to be attributed to the rather limited understanding of the tendon's inner material properties (Kuo et al., 2010), in particular with respect to its viscoelastic properties (Ganghoffer et al., 2016). The latter depend both on the physiology and on the loading type applied (e.g., quasi-static, high strain rate) (Oftadeh et al., 2018; Wu et al., 2018; Kuznetsov et al., 2019). The use of biocompatible materials, which mimic the physiological functionality of the native tissue constitutes a key aspect for any tendon restoration process (Ganghoffer et al., 2016). During the last years, substantial efforts have been devoted to the engineering of novel biomaterials with enhanced structural performance and improved biochemical compatibility (Zhang et al., 2017; Li et al., 2018a,b; Li S. et al., 2018; Liu et al., 2018). However, primal tendon inner material attributes, such as the effective viscosity at the tendon fiber scale remain unquantified.

In the current work, numerical models that describe the tendon's fascicle and fiber scale mechanics are combined with probabilistic inference models and experimental observations. By that means, a quantitative link between the tendon's viscoelastic mechanical response at the fascicle scale and experimental data is established, through a Bayesian inference framework (Papadopoulos et al., 2012; Taflanidis and Beck, 2013; Farrell et al., 2015). This allows for the quantification of uncertain tendon inner mechanical parameters that determine the experimentally observed fascicle-scale mechanics. The paper is structured as follows: in section Materials and Methods the parametrization of the fascicle's viscoelastic models is provided and the mathematical formalism of the Bayesian probabilistic framework is discussed (section Inference of the Fascicle's Viscoelastic Mechanical Properties), summarizing the experimental data used for the study (section Tendon Fascicle Experimental Data). In section Results, the results of the inference process are provided, namely the elastic and viscous fiber-scale material properties, which can reproduce the experimental observations reported at the fascicle scale. The work continuous with an overall discussion of the obtained results and a concluding summary in section Discussion and Conclusions.

## Materials and Methods

### Tendon Fascicle Geometry and Mechanical Models

We describe the fascicle's elastic and viscoelastic relaxation response in a two-step process. More specifically, we compute its elastic response with a dedicated composite helical fascicle finite element model, which is comprised of fibers immersed in a matrix substance, detailed in Karathanasopoulos et al. (2017). The fascicle geometry follows a helical angle θ, with respect to the plane perpendicular to the tendon evolution, as schematically depicted in Figure 3A. The fascicle contains fibers in different fiber contents *f*_*r*_ defined as the ratio of the area *A*_*f*_ covered by fibers (dark-gray) over the total fascicle cross sectional area *A*_*t*_ (Figure 3B). We allow for the composition of fascicles to vary so that different fiber contents are captured. More specifically, in order to take into consideration a large part of the experimentally observed fascicle compositions (Goh et al., 2008; Hansen et al., 2010; Svensson et al., 2012), we build fascicle models with fiber contents of 35% up to 65% with a spacing of 5%, accounting for a fiber swelling in the determination of the upper content limit (Hansen et al., 2010). For the linear finite element computations, the fascicles' domain covered by fibers is assigned a Young's modulus *E*_*f*_, while the matrix a modulus *E*_*m*_. The model computes the effective fascicle modulus *E*_*fasc*_ and the fascicle's volumetric behavior ν_*fasc*_ = Δ*R*/*R*/ε_*z*_ (Figure 3C) for different fiber content values *f*_*r*_ and angular arrangements θ (Figure 3A), which are considered to define distinct fascicle model classes $M_{f_{r}}^{\theta }$. Both parameters are complex functions (*f*) of the tendon fascicle's inner material and geometric properties *f*(*f*_*r*_, θ, *E*_*f*_, *E*_*m*_) (Reese et al., 2010, 2013; Karathanasopoulos et al., 2017). We enumerate a total of 49 fascicle model classes ${\left\{M\right\}}_{i=1}^{49}=M_{f_{r}}^{\theta }$ each *i* uniquely referring to a pair $\left(f_{r},\theta \right),f_{r}=35\ldots 65\%,\theta =70^{o}\ldots 76^{o}$. The reader is referred to Karathanasopoulos and Hadjidoukas (2016) and Karathanasopoulos et al. (2017) for a detailed description of the numerical modeling specifications and for a quantification of the associated computational cost.

Following the linear computation, the relaxation response of the tendon fascicle is computed, using a Maxwell standard linear solid model (Figure 2). The time-dependent relaxation curve is characterized by a relaxation experiment, using a single viscosity parameter η, as follows (Christensen, 1982; Lakes, 2009; Shen, 2010):

(1) $$\begin{array}{l} E_{fasc}\left(t\right)=f_{r}\left(E_{\infty }+E_{R}e^{-\frac{E_{R}}{\langle \eta \rangle }t}\right)+\left(1-f_{r}\right)E_{m}\left(t\right) \end{array}$$

where in Equation (1), *E*_*R*_ is equal to the fiber's elastic modulus loss *E*_*R*_ = *E*_*f*_ (*t* = 0) − *E*_∞_ during the relaxation process (Figure 2). For *t* = 0, the fascicles's elastic modulus is equal to its linear, non time-dependent value, so that the previous relation entails *E*_*fasc*_(*t* = 0) = *f*_*r*_(*E*_*R*_+*E*_∞_) + (1−*f*_*r*_)*E*_*m*_(*t*). Noting that the matrix modulus *E*_*m*_ has been shown to be more than two order of magnitudes lower than the one of its fibrillar components (*E*_*f*_ > > *E*_*m*_) (Ault and Hoffman, 1992; Reese et al., 2010; Karathanasopoulos et al., 2017), the primal contributors in the fascicle's relaxation curves (Figure 3D) described by Equation (1) are its embedded fibers (*E*_*m*_ (*t*) ≈ 0). As a result, the fascicle's time-dependent modulus loss is essentially characterized by the relaxation behavior of its inner, matrix-embedded fibers, so that the viscous parameter 〈η〉 entering Equation (1) describes the effective -homogenized- viscosity of its embedded fibers, here named as η_*f*_.

We subsequently parametrize each fascicle model class $M_{f_{r}}^{\theta }$ by a total of three parameters, namely by the elastic modulus of the fiber and of the matrix *E*_*f*_ and *E*_*m*_ and by the viscous modulus of its embedded fibers η_*f*_. We note that the fascicle's effective Poisson's ratio value ν_*fasc*_ is a non-linear function of its fiber content *f*_*r*_, angle θ (thus $M_{f_{r}}^{\theta }$) and fiber and matrix elastic moduli values *E*_*f*_ and *E*_*m*_, as elaborated in Appendix section Fascicle Poisson Ratio. Accordingly, the fascicle's time-dependent response well differs, depending on the combination of the elastic and viscous properties entering Equation (1), as demonstrated in Appendix section Fascicle Relaxation Response. Using the previously defined parameters, we compute a total of three quantities of interest for each model class $M_{f_{r}}^{\theta }$, as follows:

(2) $$\begin{array}{rcl} E_{fasc}^{0} & = & E_{fasc}^{}\left(t=0\right)=q_{1}\left(\varphi |M_{f_{r}}^{\theta }\right),\;\nu_{fasc}^{0}=q_{2}\left(\varphi |M_{f_{r}}^{\theta }\right),\\ {Ē}_{fasc}\left(t\right) & = & q_{3}\left(\varphi |M_{f_{r}}^{\theta }\right) \end{array}$$

where in Equation (2), Ē_*fasc*_(*t*) stands for the time-evolution of the normalized fascicle modulus, the normalization carried out with respect to its initial elastic modulus $E_{fasc}^{0}$.

### Inference of the Fascicle's Viscoelastic Mechanical Properties

The goal in parameter inference is to infer the parameters $\varphi \;\in \;{\text{R}}^{N_{\varphi }}$ after observing the data *D* = {*d*_*i*_|*i* = 1, …, *N*}. The computational model used to simulate the data *D* can be described through a function *F*(*x*; φ) ∈ R^*N*^, which depends on both the parameters φ and on input parameters $x\in {\text{R}}^{N_{x}}\;$ that are not being inferred.

In Uncertainty Quantification we are interested in sampling the conditional posterior distribution *p*(φ|*D*), using the Bayes' theorem, defined as follows:

(3) $$\begin{array}{r} p\left(\varphi |D,\text{M}\right)=\frac{p\left(D|\varphi ,\text{M}\right)p\left(\varphi |\text{M}\right)}{p\left(D|\text{M}\right)} \end{array}$$

where in Equation (3) *p*(*D*|φ, M) is the likelihood function, *p*(φ|M) is the prior probability distribution and *p*(*D*|M) is the model evidence. Here, M stands for the model under consideration and contains all the information that describes the computational and the statistical model. The models are here parametrized by the fascicle angle θ and fibrillar content *f*_*r*_, denoted as $M_{f_{r}}^{\theta }$, as explicated in section Tendon Fascicle Geometry and Mechanical Models. The likelihood function is a measure of the probability that the data *D* are reproduced by the computational model employed, while the prior probability encodes all available information before observing any data. In the present work, we make the assumption that the data *D*_*i*_ are independent and normally distributed around the observable of the model, with a proportional model error, so that:

(4) $$\begin{array}{c} D_{i}=F_{i}(x;\varphi )+F_{i}(x;\varphi )\varepsilon ,\varepsilon \overset{\tilde{\;}}{}N(0,\sigma_{n}^{2}) \end{array}$$

where the data *D*_*i*_ are assigned a proportional error model. Using Equation (4), the likeli- hood function *p*(*D*|φ, M) takes the form:

(5) $$\begin{array}{rcl} p\left(D|\varphi ,\text{M}\right) & = & N\left(D|F\left(x,\varphi \right),\text{Σ}\left(x,\vartheta \right)\right),\\ \text{Σ}\left(x,\vartheta \right) & = & \sigma_{n}^{2}diag\left(F\left(x,\varphi \right)\right) \end{array}$$

Where the model and the error parameters can be described by the vector $\vartheta ={\left(\varphi^{T},\sigma_{n}\right)}^{T}$ in a unified form. In most practical applications the posterior distribution (Equation 3) cannot be expressed analytically. Moreover, the model evidence *p*(*D*|M) is typically not known, since it is given by integration of the nominator of Equation (3) over the potentially high dimensional domain of the parameters. In the current work, we rely on efficient sampling algorithms to identify the posterior distribution. In particular, we use the Transitional Markov Chain Monte Carlo algorithm (TMCMC) (Ching and Chen, 2007; Farrell et al., 2015). The algorithm starts by sampling the prior distribution, which is usually trivial to be sampled, and through annealing steps it provides samples from the posterior distribution. One major advantage of this algorithm is its ability to sample multimodal distributions and provide low bias estimators of the model evidence (Ching and Chen, 2007).

**Figure 4:**
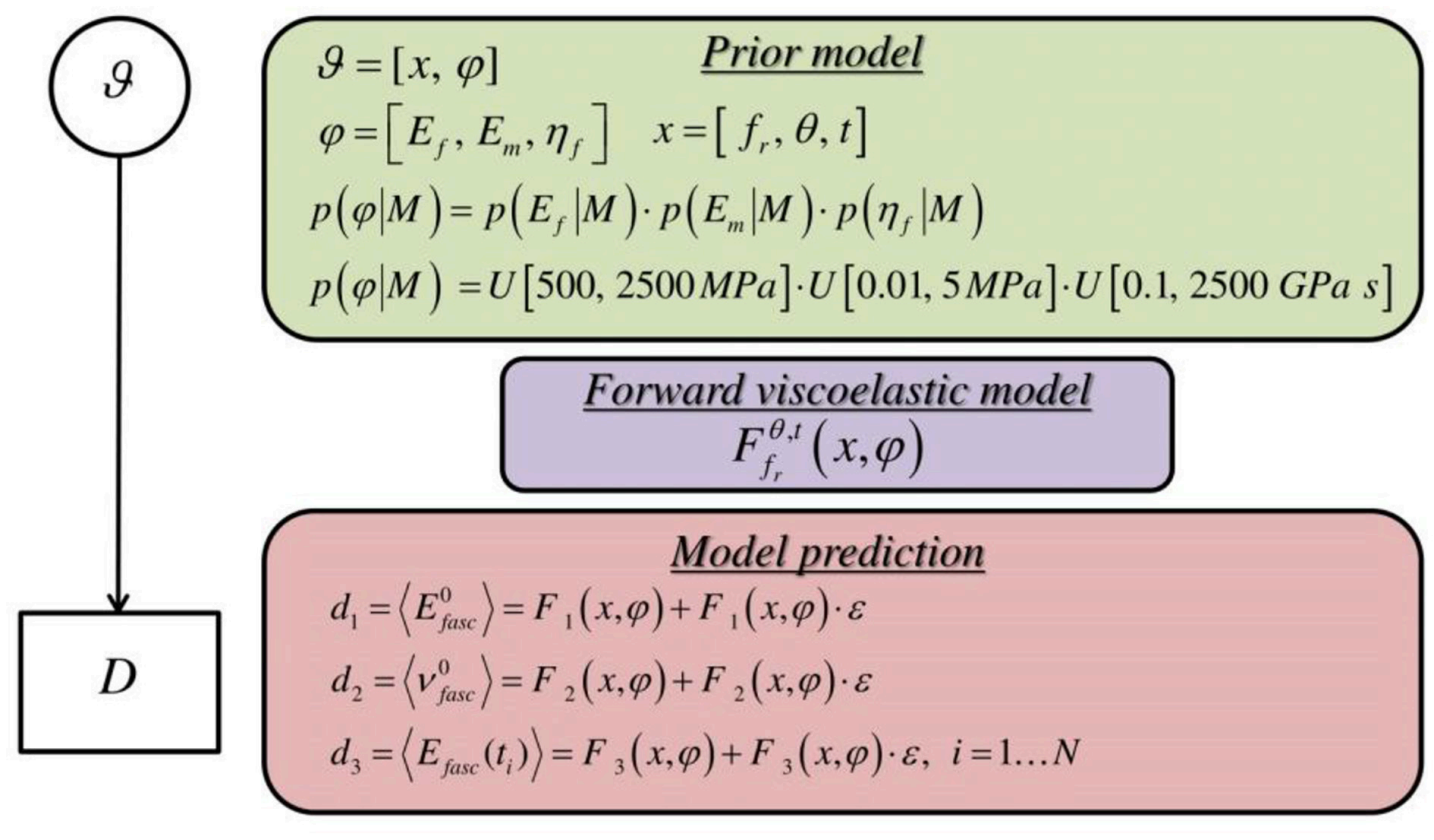
Graphical probability model describing the formulation of the forward stochastic model used to reproduce the data D.

Here, we make use of uniform prior distributions *U* for each of the modeling parameters φ = [*E*_*f*_, η_*f*_, *E*_*m*_] which encompass and exceed the range reported in the corresponding experimental observations of section Introduction, indicating prior ignorance with respect to their actual value. In particular, we allow their values to vary in between 500−2500 *MPa*, 0.1−2500 *GPa s* and 0.01−5 *MPa*. A graphical representation of the probability model formulation is provided in Figure 4.

### Tendon Fascicle Experimental Data

For the inference process we combine multiple available experimental data, pertaining to both the linear and the time-dependent fascicle response. In particular, for the quantities of interest *q*_1_ and *q*_2_ of Equation (2), we made use of the elastic, non time-dependent experimental data reported in Derwin and Soslowsky (1999), Haraldsson et al. (2009) and Svensson et al. (2010); and Lynch (2003) and Cheng and Screen (2007), accordingly. What is more, for the time-dependent quantity *q*_3_ of Equation (2), we digitalize the experimental relaxation curves provided in Screen (2008) and Davis and De Vita (2012) and summarized in Figure 3D, using ~30 equally spaced time-intervals along the relaxation curve, as control points for our modeling predictions. We consider a total of five groups of experimental observations *D*_1_, *D*_2_, *D*_3_, *D*_4_, and *D*_5_. Each data set *D*_*i*_ contains a set of normalized fascicle moduli ratios Ē_*fasc*_(*t*), as schematically depicted in Figure 3D. In Table 1, we summarize the considered experimental measurements for each group of experimental observations.

**Table 1:** Fascicle experimental data sets *D*_*i*_ encompassing the fascicle initial elastic modulus *E*_*fasc*_, Poisson's ratio value ν_*fasc*_, and the time-dependent response of its modulus Ē_*fasc*_(*t*).

	***E*_*fasc*_(*t* = 0) (Derwin and Soslowsky, 1999; Haraldsson et al., 2009; Svensson et al., 2010)**	****ν_*fasc*_(−)****	****Ē_*fasc*_(*t*)(−)****
*D*_1_	640|480|550	1 (Cheng and Screen, 2007)	Figure 3D (Sc) (Screen, 2008)
*D*_2_	640|480|550	3 (Lynch, 2003)	Figure 3D (Sc) (Screen, 2008)
*D*_3_	640|480|550	1 (Cheng and Screen, 2007)	Figure 3D (Dv_1_) (Davis and De Vita, 2012)
*D*_4_	640|480|550	1 (Cheng and Screen, 2007)	Figure 3D (Dv_2_) (Davis and De Vita, 2012)
*D*_5_	640|480|550	1 (Cheng and Screen, 2007)	Figure 3D (Dv_3_) (Davis and De Vita, 2012)

## Results

**Figure 5:**
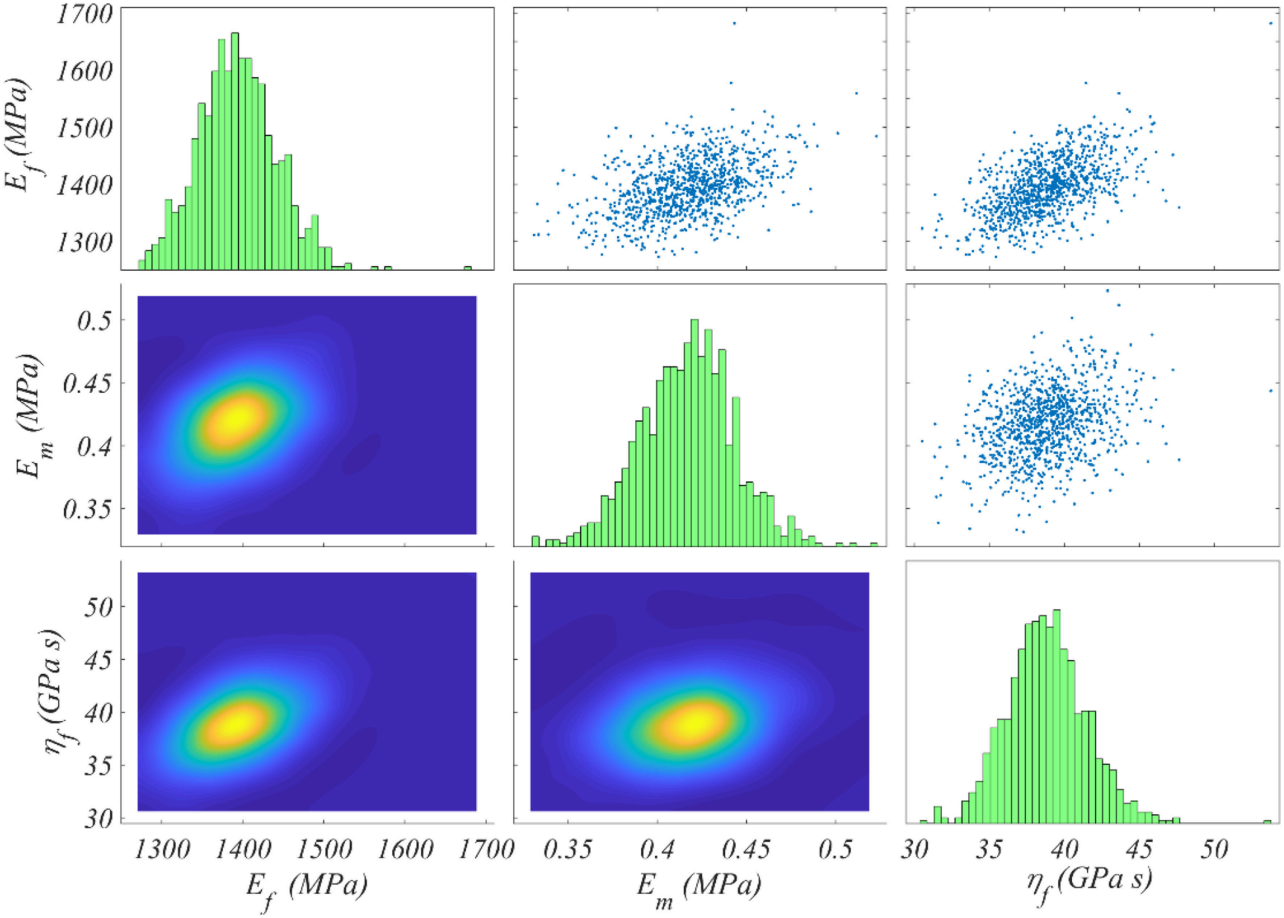
Posterior frequency distribution of the modeling parameters φ for a model $M_{f_{r}}^{\theta }$ with a content *f*_*r*_ = 35% and an angle θ = 75^*o*^. The posterior distribution corresponds to the data set *D*_1_ of Table 1.

We compute the posterior sample distributions using the TMCMC algorithm elaborated in Ching and Chen (2007) for all model classes $M_{f_{r}}^{\theta }$, for each of the data sets *D*_*i*_ included in Table 1. In Figure 5 we provide the posterior frequency distribution for each of the inferred parameters φ = [*E*_*f*_, η_*f*_, *E*_*m*_] for the model $M_{f_{r}}^{\theta }$ with a fiber content *f*_*r*_ = 35% and an angle θ = 75^*o*^. The results correspond to the data set *D*_1_ of Table 1.

Figure 5 depicts a posterior distribution of the modeling parameters φ with a clear clustering of values for each of the *E*_*f*_, η_*f*_, *E*_*m*_. In particular, for each parameter, the range of values with a non-zero posterior probability is a narrow subspace of the uniform prior used as initial modeling hypothesis and summarized in Figure 4. More specifically, the fiber modulus *E*_*f*_ ranges in between 1,300 and 1, 500 *MPa*, while the matrix modulus *E*_*m*_ within 0.35 and 0.5 *MPa*. Accordingly, the entire probability mass for the viscosity parameter η_*f*_ ranges among 35 and 45 *GPa s*. The model proportional error (Equation 4) associated to the results of Figure 5 is restrained to a maximum of 8% for all model classes and data sets *D*_*i*_. Analogous posterior parameter frequency distributions with the one provided in Figure 5 are obtained for all model classes $M_{f_{r}}^{\theta }$ and data sets *D*_*i*_ of Table 1.

**Figure 6:**
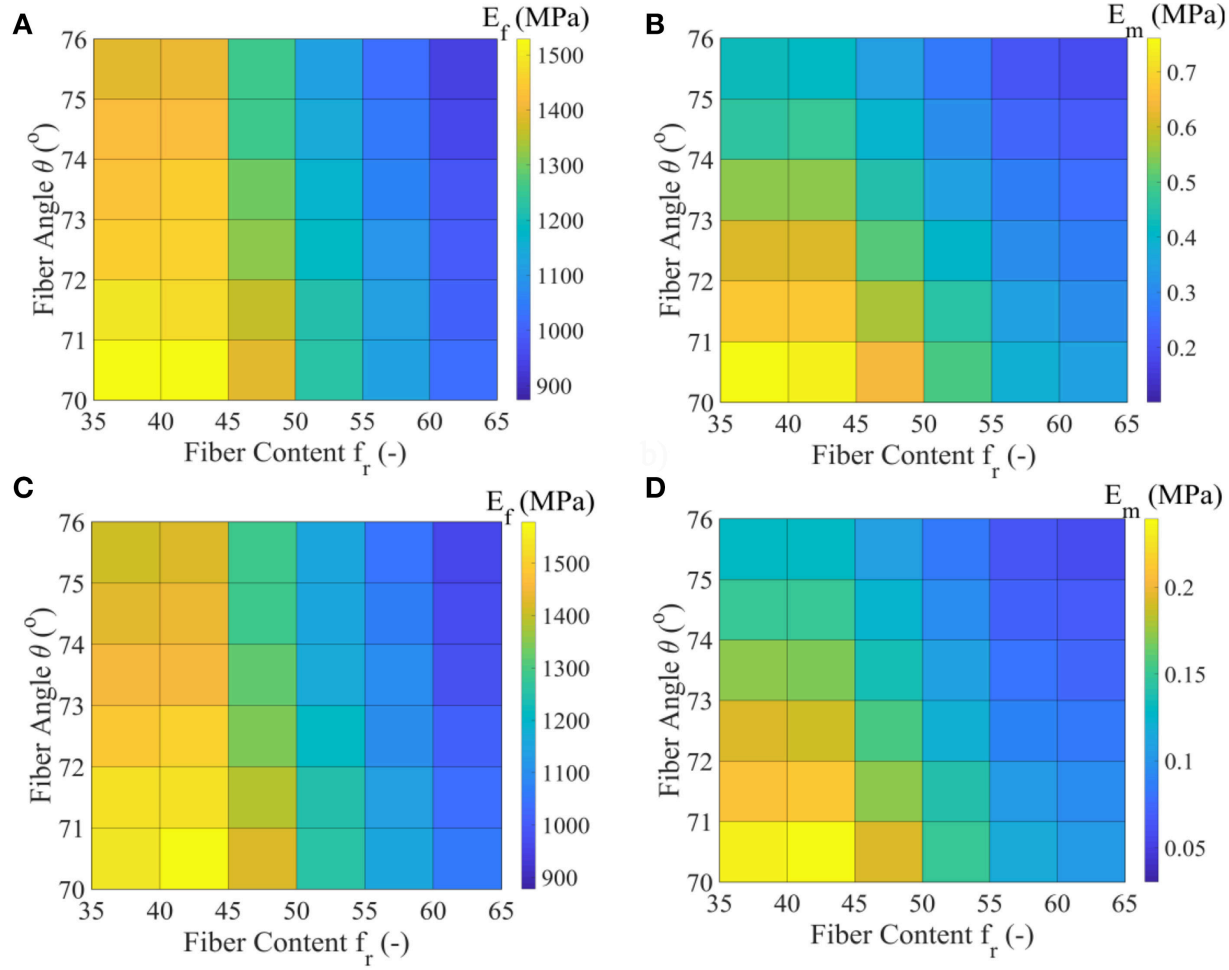
Maximum A-Posteriori (MAP) values for the fiber elastic modulus *E*_*f*_ and matrix elastic modulus *E*_*m*_ for the all model classes of the data set *D*_1_ (subplots **A,B**) and data set *D*_2_ (subplots **C,D**) of Table 1.

In Figures 6A,B we provide the values *E*_*f*_ and *E*_*m*_ that maximize the posterior PDF for each respective model class $M_{f_{r}}^{\theta }$ for the data set *D*_1_. These values, named as the Maximum A-Posteriori (MAP) values are defined as $\left(E_{f},E_{m},\eta_{f}\right)=\arg\max_{\varphi }F\left(\varphi |D,M_{f_{r}}^{\theta }\right)$. In Figures 6C,D, we provide the values for *E*_*f*_ and *E*_*m*_ for the data set *D*_2_, pertaining to a fascicle Poisson's ratio value ν = 3 (Table 1).

Figures 6A,C suggest a non-linear relation between the fiber modulus *E*_*f*_ and the fiber content *f*_*r*_, so that the higher the fiber content value, the lower the most probable fiber modulus value obtained. In particular, a maximum and a minimum most probable fiber modulus of approximately 1550 *MPa* and 900 *MPa* is obtained for a 35% and a 65% fiber content, accordingly. The fiber angle θ affects the magnitude of the *E*_*f*_ value: higher angle values θ correspond to lower fiber moduli *E*_*f*_, irrespective of the fascicle's fibrillary content *f*_*r*_. The associated difference lies however within a maximum range of 150 *MPa* for a given fiber content value (Figures 6A,C).

The different fascicle Poisson's ratio values (ν_*fasc*_ in Table 1) used among the data sets *D*_1_ and *D*_2_, appear to minorly affect the peaks of the posterior probability distribution for *E*_*f*_ (Figures 6A,C), while they decisively affect the *E*_*m*_ MAP values (Figures 6B,D). In particular, the higher fascicle Poisson's ratio value of data set *D*_2_ results in considerably reduced *E*_*m*_ MAP values with respect to the ones computed for the data set *D*_1_. However, the dependence of *E*_*m*_ on *f*_*r*_ and θ is analogous to the one obtained for the fiber modulus *E*_*f*_. The a-posteriori, most probable inferred fiber moduli values *E*_*f*_ well compare to the ones suggested by the experimental study of Gentleman et al. (2003), while the matrix modulus values *E*_*m*_ to the analytical modeling predictions of Ault and Hoffman (1992), being smaller than 0.7 *MPa* in the entire parametric space. For the data sets *D*_3_ to *D*_5_ of Table 1, fiber and matrix moduli values that differ by a maximum of 5% with respect to the ones provided in Figures 6A,B are obtained. The inferred MAP values for all data sets *D*_*i*_ and model classes $M_{f_{r}}^{\theta }$ are provided in the form of Supplementary Material.

**Figure 7:**
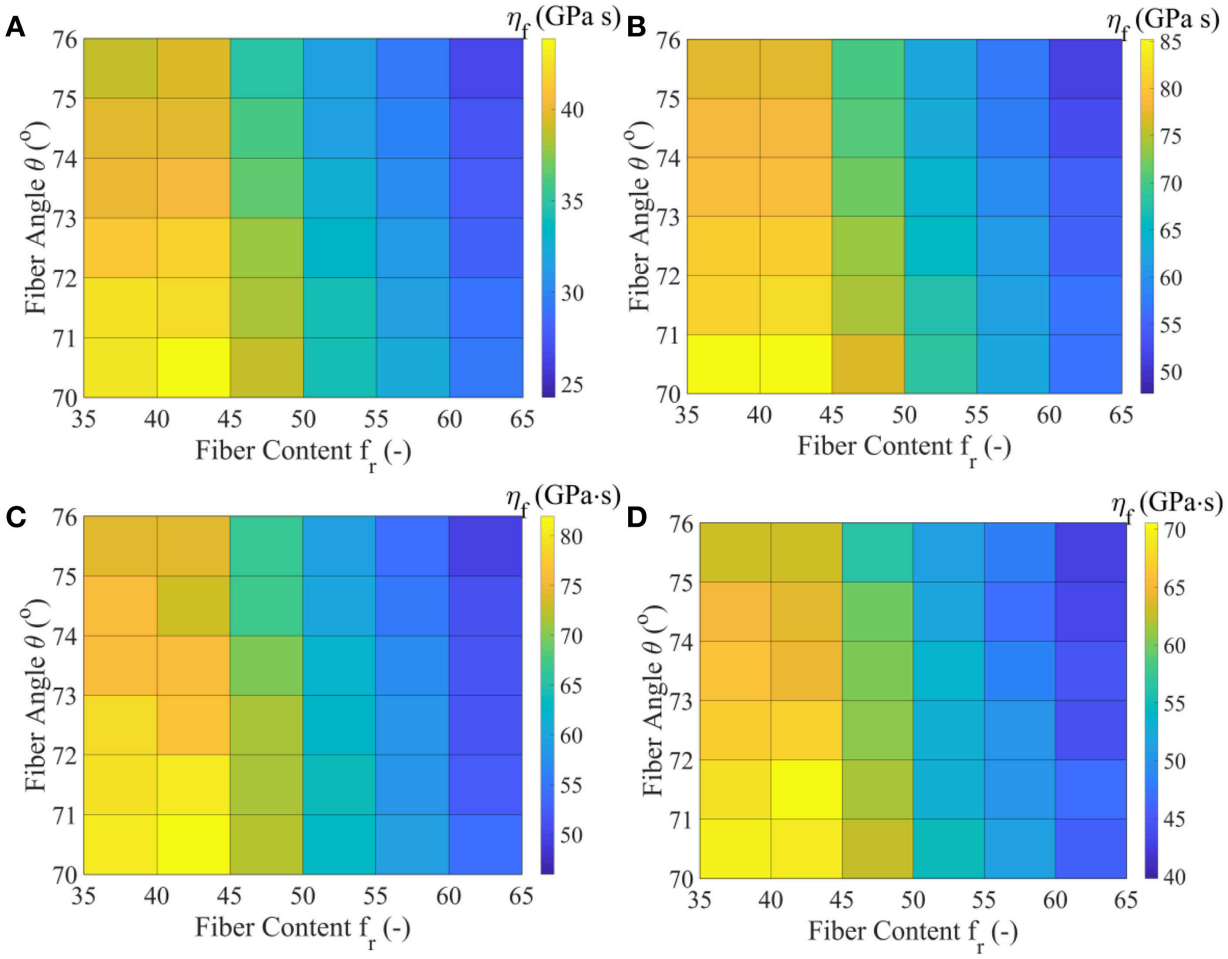
MAP values for the effective viscous modulus η_*f*_ for all model classes $M_{f_{r}}^{\theta }$ acquired for the data-sets *D*_1_ **(A)**, *D*_3_ **(B)**, *D*_4_ **(C)**, and *D*_5_ **(D)**, as defined in Table 1.

In Figure 7, we provide the MAP values for the viscoelastic modulus η_*f*_ of the matrix-embedded fibers for all model classes $M_{f_{r}}^{\theta }$ introduced in section Inference of the Fascicle's Viscoelastic Mechanical Properties, corresponding to the relaxation curves of Figure 3D. The maximum a-posteriori viscosity values are provided for the data sets *D*_1_, *D*_3_, *D*_4_, and *D*_5_ of Table 1.

Figure 7 suggests relaxation moduli values η_*f*_ for the embedded fibers of several tens of *GPa s* for all model classes $M_{f_{r}}^{\theta }$ and data sets *D*_*i*_. In particular, a minimum most probable value of approximately 25 *GPa s* is obtained for the highest fiber content and fiber angle model $M_{65}^{76}$. Data set *D*_3_ yields the maximum viscosity parameter η_*f*_ = 85 *GPa s* for the lowest fibrillar content and fiber angle model class analyzed $M_{35}^{70}$. The relaxation curves corresponding to data-sets *D*_4_ and *D*_5_ of Table 1 relate to a range of η_*f*_ values in between 40 and 80 *GPa s*. For all data sets, low fibrillar contents *f*_*r*_ pair to high relaxation moduli values, while increased fiber angles θ to lower η_*f*_ values for a given data set *D*_*i*_ and content value *f*_*r*_. We note that the linear elastic, non time-dependent fascicle Poisson's ratio value ν_*fasc*_ is independent of the relaxation moduli η_*f*_, so that no significant differences arise between the inferred η_*f*_ values of data sets *D*_1_ and *D*_2_. The inferred MAP values for the parameter [*E*_*f*_, η_*f*_, *E*_*m*_] are provided for all data sets *D*_*i*_ and model classes $M_{f_{r}}^{\theta }$ in the form of Supplementary Material. Each set of material properties provided in Figures 6, 7 can well-reproduce both the elastic and the experimentally observed time-dependent fascicle behavior summarized in Table 1.

**Figure 8:**
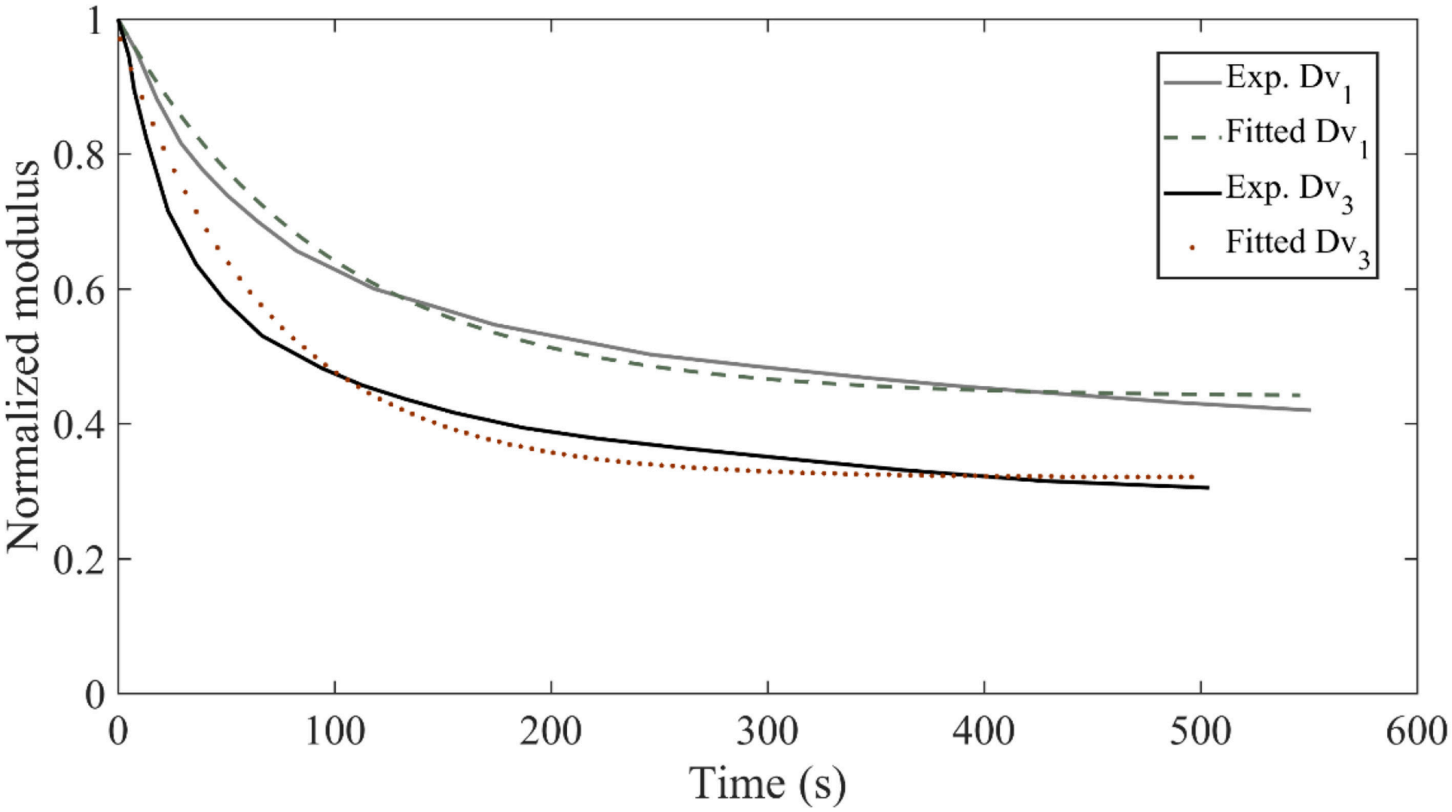
Relaxation response for the experimental curves *Dv*_1_ and *Dv*_3_ of Figure 3D compared to the relaxation response obtained with the inferred mechanical properties of the data sets D_3_ and D_5_ for the model class $M_{35}^{70}$.

In Figure 8, we provide for verification purposes a comparison of the fascicle relaxation response as computed by the previously inferred mechanical parameters and by the experimentally provided response. To that scope, we make use of the inferred MAP elastic and viscous mechanical properties part of them depicted in Figures 6C,D, 7B,D for the model class $M_{35}^{70}$, which we compare with the experimental curves *Dv*_1_ and *Dv*_3_, provided in Figure 3D.

Figure 8 suggests a very good agreement between the experimental relaxation response and the relaxation behavior arising from the inferred mechanical properties. Analogous behavior is obtained for all model classes $M_{f_{r}}^{\theta }$ and data sets *D*_*i*_ of Table 1.We note that non-optimized sets of the parameters *E*_*f*_, *E*_*m*_, η_*f*_ lead to utterly different fascicle relaxation behaviors, as elaborated and showcased in Appendix section Fascicle Relaxation Response.

## Discussion and Conclusions

Knowledge of the tendon's inner material properties is a primal prerequisite for the application of any successful treatment or restoration process (Snedeker and Foolen, 2017; Karathanasopoulos et al., 2019). However, experimental data on the material attributes of inner, lower tendon subunits are commonly highly uncertain, while fundamental parameters, such as the η_*f*_ of tendon fibers remain unquantified (section Introduction). Combining upper (fascicle) and lower (fiber) tendon scale mechanics with experimental observations, provides a means to infer and quantify lower scale tendon mechanical properties, so that they are able to reproduce upper scale mechanics.

The inferred fiber scale mechanics indicate that the most probable fiber modulus values *E*_*f*_ range between 900 and 1, 600 *MPa* (Figure 6), thus in a subspace of the literature reported range of moduli (Kato et al., 1989; Gentleman et al., 2003), when using experimental observations at the fascicle tendon scale. In particular, if information on the fibrillar content value *f*_*r*_ is provided, the fiber modulus *E*_*f*_ can be estimated, within a range of 200 *MPa* when the 95% of the mass of the posterior probability distribution is accounted for (Figure 5). Moreover, for the observed volumetric behaviors at the fascicle scale to be retrieved (Table 1), a matrix modulus *E*_*m*_ below 1 *MPa* is required for all fibrillar content and fiber angle values model classes $M_{f_{r}}^{\theta }$. Increased fascicle lateral volumetric contractions are obtained by decreasing the matrix modulus *E*_*m*_ (Figure 6), thus by increasing the relative contrast of elastic properties *E*_*f*_/*E*_*m*_ at the fiber scale.

The viscous moduli η_*f*_ values provided in Figure 7 constitute the first quantitative estimates of the embedded fiber viscosity that is based on experimental observations. For a given fascicle composition $M_{f_{r}}^{\theta }$ and data set *D*_*i*_, the posterior distribution of the embedded fiber viscosity suggests η_*f*_ values which at most differ by 10 *GPa s* (Figure 5), considering the entire mass of the posterior probability distribution. For fascicles with a low fibrillar content and fiber angle, most probable η_*f*_ values of more than 40 *GPa s* are obtained, irrespective of the data-set *D*_*i*_ considered (Figure 7). The values of Figure 7 combined with the most probable fiber elastic moduli values of Figure 6 can be viewed as a fundamental reference table in the design of artificial tendons. In particular, each pair of most probable *E*_*f*_, η_*f*_ values can be used as a set of basic elastic and time-dependent material properties for the engineering of artificial fibers in scaffold-based tendon restoration processes (Kuo et al., 2010; Abdullah, 2015; Sandri et al., 2016).

**Figure 9:**
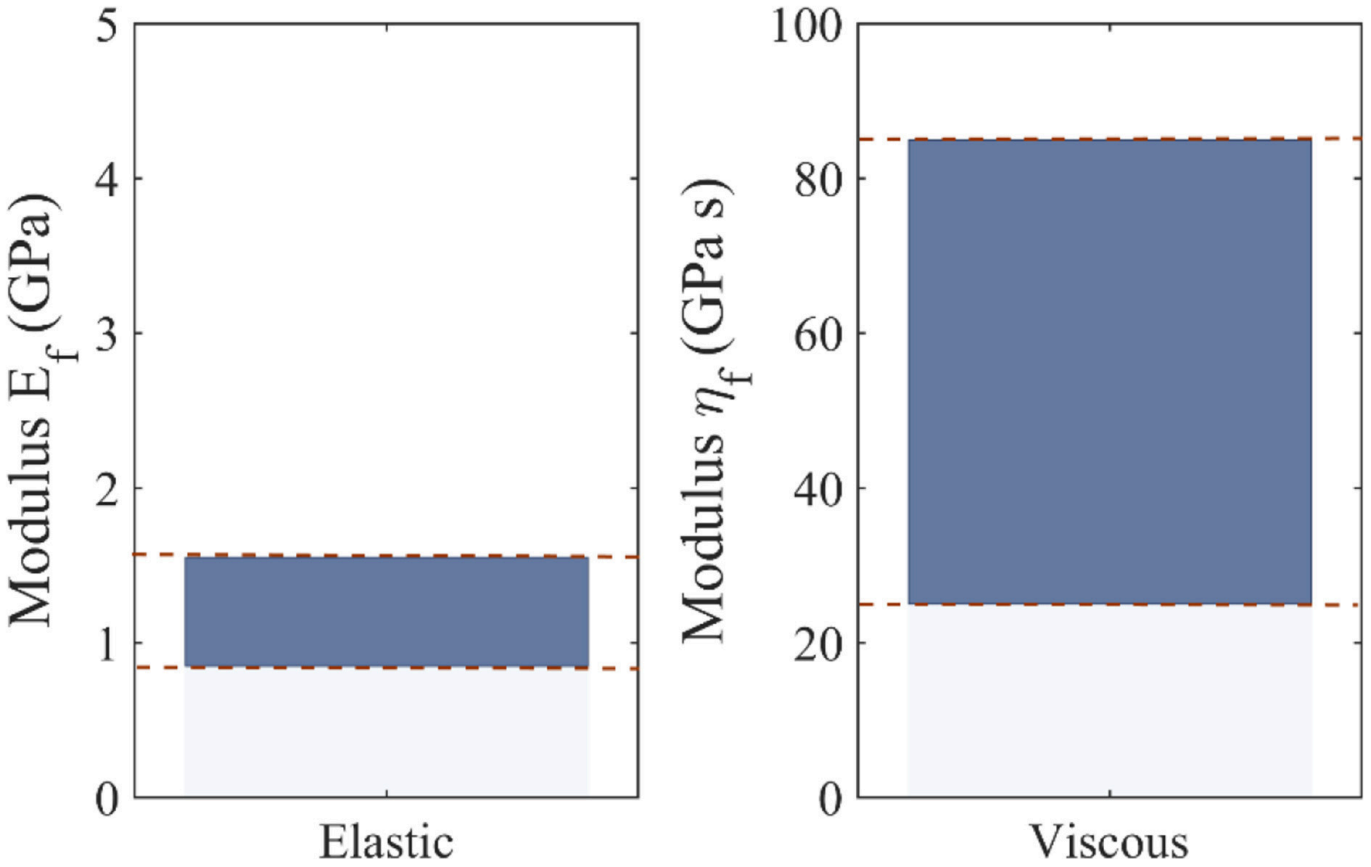
Range of most probable elastic *E*_*f*_ and viscous fiber η_*f*_ moduli values, including all possible fiber content values *f*_*r*_, fiber orientations θ, and data-sets *D*_*i*_.

Note that the differences observed among the most probable viscoelastic embedded fiber moduli values η_*f*_ (Figure 7) reflect to a large extent the discrepancies among the experimentally reported relaxation curves of Figure 3, as well as the wide range of possible fibrillar content values *f*_*r*_. Figure 9 summarizes the range of values for the most probable elastic and viscous fiber parameters (Figures 6, 7), when all fiber content values *f*_*r*_, fiber angles θ, and data-sets *D*_*i*_ are considered. In order to further delimit the range of probable elastic and viscous parameters, further relaxation experiments need to be conducted at the tendon fascicle scale. The latter need to provide mean and standard deviation values for the relaxation moduli at different time-frames throughout the relaxation process at different strain magnitudes and initial loading strain-rates. Data of the kind will allow for a series of secondary analysis to be carried out, which are as of now intractable. In particular, they will allow for the consideration of more complex relaxation models with multiple relaxation times or for the modeling of strain-rate and strain-magnitude effects (Oftadeh et al., 2018). Moreover, they would allow to account for the presence of geometric or material non-linearities, phenomena which typically play a role in the mechanical behavior of biological materials (Kuznetsov et al., 2019); objectives which are way beyond the current tendon mechanics-related data availability.

## Author Contributions

NK: conception, design, results acquisition, and analysis. J-FG: modeling, analysis, and interpretation of results.

### Conflict of Interest Statement

The authors declare that the research was conducted in the absence of any commercial or financial relationships that could be construed as a potential conflict of interest.

## Figures and Tables

**Figure A1:**
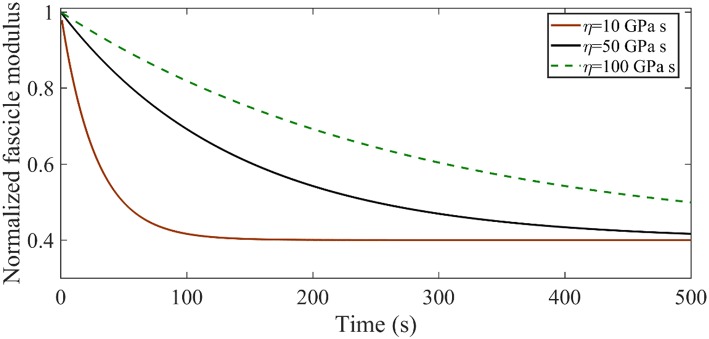
Relaxation response for an initial elastic modulus *E*_*fasc*_ = 600 *MPa* with a relaxing elastic modulus *E*_*R*_ = 720 *MPa* for different viscosity values η.

**Figure A2:**
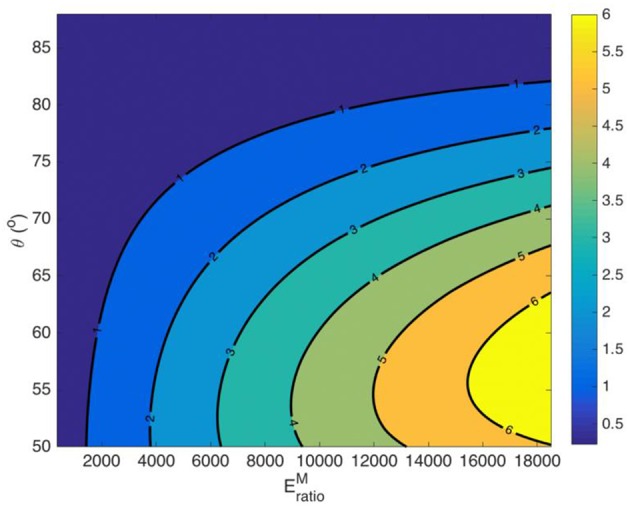
Fascicle effective Poisson ratio values for a fiber content *f*_*r*_ = 0.6 and a matrix modulus *E*_*m*_ = 1 *MPa*.

## Supplementary Material

Data Sheet 1 Summary of the MAP values for the datasets D_1_–D_5_ of Table 1.

## Appendix

### Fascicle Relaxation Response

The time-dependent relaxation response of tendon fascicles depends on both their elastic properties *E*_∞_, *E*_*R*_ as indicated by Equation 1 on their viscosity η_*f*_. In Figure A1 we provide an insight in the role of the effective viscosity parameter η_*f*_ on the form of the relaxation curve. To that scope, we compute the relaxation response of a tendon fascicle with an initial zero-time (*t* = 0) elastic modulus *E*_*fasc*_(0) = 600 *MPa* (with an *E*_*f*_ = 1025 *MPa* *E*_*m*_ = 0.4 *MPa* *f*_*r*_ = 0.65 θ = 72^*o*^) and a relaxing elastic modulus *E*_*R*_ = 720 *MPa* for viscosity values of η_*f*_ = 10 *GPa s* η_*f*_ = 50 *GPa s* and η_*f*_ = 100 *GPa s*.

Figure A1 suggests that increasing the viscosity parameter η results in higher time-dependent moduli values *E*_*fasc*_(*t*), with the maximum difference to amount to several hundreds of *MPa*. As an example, for η = 10 *GPa s* at *t* = 100*s* the structure has relaxed to *E*_*fasc*_(100) = 240 *MPa*, while for η = 50 *GPa s* the corresponding value is *E*_*fasc*_(100) = 420 *MPa*.

### Fascicle Poisson Ratio

The effective Poisson ratio of the fascicle ν_*fasc*_ hinges on both the material properties of the fiber and the matrix (*E*_*f*_, *E*_*m*_) and on its geometric arrangement (*f*_*r*_, θ) [21, 25]. As a result, different volumetric fascicle behaviors are obtained depending on the set of parameters selected. In Figure A2, we provide the effective Poisson's ratio values for a case study with a fibrillar content *f*_*r*_ = 0.6 over different helix angles θ and for different ratios of the fiber modulus to the matrix modulus $E_{ratio}^{M}=E_{f}/E_{m}$, computed for a matrix modulus value of *E*_*m*_ = 1.

Figure A2 suggests a highly non-linear relation between the moduli ratio value $E_{ratio}^{M}$ and the fascicle's Poisson's ratio value, which depends on both the fibrillar content *f*_*r*_ and on the fiber angle θ.
